# Powder Bed Thermal Diffusivity Using Laser Flash Three Layer Analysis

**DOI:** 10.3390/ma16196494

**Published:** 2023-09-29

**Authors:** Ummay Habiba, Rainer J. Hebert

**Affiliations:** 1 Department of Materials Science and Engineering, University of Connecticut, Storrs, CT 06269, USA; 2 Pratt & Whitney Additive Manufacturing Center, University of Connecticut, Storrs, CT 06269, USA; 3 Institute of Materials Science, University of Connecticut, Storrs, CT 06269, USA

**Keywords:** additive manufacturing, laser powder bed fusion (LPBF), thermal diffusivity, laser flash technique, powdered sample, powder cell, three layer analysis, INCONEL718 solid, INCONEL718 powder, Ti64 powder

## Abstract

The thermal diffusivity of powder bed plays a crucial role in laser powder bed fusion (LPBF) additive manufacturing. The mechanical properties of the parts built by LPBF are immensely influenced by the thermal properties of the powder bed. This study aims to measure the thermal diffusivity of metallic powder, nickel-based super alloy Inconel718 (IN718), in LPBF using laser flash three-layered analysis in a DLF1600 instrument, which incorporates a special powder cell to encapsulate the powdered sample. Measurements were performed at different temperatures. The thermal diffusivity of several reference samples was measured for the purpose of validating the test results, and it was compared to published data for identical measures. It was observed that experimental results for powder samples were smaller than the actual thermal diffusivity of the sample. R software analysis was used to analyze test data in order to obtain powder thermal diffusivity values that were close to the actual values.

## 1. Introduction

Additive manufacturing (AM) is an advanced manufacturing technique, which produces parts by layering material in specific patterns [1,2]. When relatively small parts with complex geometry are required, the AM community resorts to laser powder bed fusion (LPBF) [3,4]. Metal or polymeric powders are used as feedstock materials during LPBF; one or more laser beams scan over specific regions of the powder bed, melting and solidifying powder particles in their path [1,4]. The laser or electron beams transfer enough energy into the powder bed to melt powder particles that then solidify into a thin solid layer. Energy transfers as heat into regions adjacent to the laser track, i.e., into solid and powder regions. Steep temperature gradients develop in response to the very localized high energy input and the small size of the melt pool on the order of 100 µm. The steep temperature gradients across powders and solid sections in return cause energy flow and temporal temperature changes that are described commonly with Fick’s laws [5]. The temperature changes and rates of heat extraction directly affect the microstructure formation and lattice structure during melt pool solidification which have significant impacts on the mechanical properties of the solidified parts. Several studies [6,7,8] have identified lattice structure as the predominant factor influencing the mechanical performance of AM parts. However, temperature changes and heat extraction also affect the powder bed. Heat conduction into the powder bed can promote diffusive processes on and between particles. A few thermophysical properties control the response of materials to imposed temperature gradients and temperature excursions into the liquid state, among them chiefly thermal conductivity, diffusivity, surface tension, and viscosity. A necessary step of modeling LPBF is therefore to know these thermophysical properties for the powder material and the processing gas.

Mechanisms of thermal conduction in solid metallic materials are based mainly on lattice dynamics [9]. Free electrons and lattice vibration both contribute to the conduction of heat in solid materials. Heat transfers through the materials randomly suffering frequent collisions with the vibrating lattice and free electron and thermal energy transports along the direction of the lattice vibration and electron. The random motion of thermal energy results in temperature gradient [9,10]. For a steady-state heat conduction, the temperature gradient can be expressed according to Fick’s first law as [9]:

(1) $$\vec{j}=-\lambda \nabla T$$

where $\vec{J}$ is the heat flux, λ is the thermal conductivity coefficient, T is the temperature. Equation (1) relates the heat flux to the temperature gradient without any time dependence. Fick’s second law relates the changes in temperature with time to the second order partial derivative of temperature with respect to the spatial coordinate [9,11]:

(2) $$\nabla \cdot \left(\lambda \nabla T\right)+g=\rho c_{p}\frac{\partial T}{\partial t}$$

Here, *g* is a source term, *ρ* the density, and *c_p_* the specific heat. Without a source term, for constant values of the thermal conductivity throughout the material, and recognizing that the thermal diffusivity is related to the thermal conductivity, density, and heat capacity as:

(3) $$\alpha =\frac{\lambda }{\rho c_{p}}$$

Fick’s second law can be rewritten as:

(4) $$\alpha \frac{\partial T}{\partial t}-∆T=0$$

where Δ denotes the Laplace operator. Thermal diffusivity is often used as a scalar quantity, implying isotropic behavior, but in general thermal diffusivity must be expressed as a tensor of second rank [12].

For homogeneous samples, Parker et al. were among the first to develop an analytical model for the laser flash diffusivity (LFD) method [13]. This laser flash diffusivity (LFD) method has grown in popularity as a technique for measuring thermal diffusivity due to its experimental simplicity, limited thermal contact resistance, accuracy, measurement time, wide temperature range, small sample size, temperature stability during measurements, [14,15,16]. In LFD measurements, samples are uniformly and very briefly irradiated with a laser pulse and an infrared detector records the voltage changes, which are proportional to temperature changes on the back sample surface. The IR detector voltage changes with time are analyzed to determine the thermal diffusivity of the sample. Parker’s original model assumed a zero heat-loss, homogeneous samples, a one-directional heat flow, isotropic thermal diffusivity, and an infinitely fast heat pulse [13]. Subsequently, Parker’s assumptions were relaxed in a series of papers. The two main modifications in the literature were finite pulse times and heat loss conditions at the sample surfaces: Clark and Taylor [17] investigated the heat loss due to radiation at high temperatures as did Cowan [18]. Their approaches differ in that Cowan used the cooling part of the temperature vs. time curve at the rear side of an irradiated sample after the irradiation pulse while Clark and Taylor use the heating portion of the signal. Cape and Lehman [19] examined finite pulse times of the laser irradiating the sample surface. For pulse durations that are short compared to a characteristic temperature rise time, Parker’s analysis holds true. Degiovanni et al. [20] investigated the laser flash method based on the assumption that the heat pulse irradiates the front surface of the sample nonuniformly. They presented a simple solution by using an average rear face sample temperature to produce time-temperature curve. They also investigated the flash method by assuming that the heat flow is nonlinear during the measurement process. Watt developed a theoretical analysis of the laser flash technique, taking nonuniform irradiation and finite pulse times into account for a cylindrical sample [21]. Since these early studies and LFD model developments, the laser flash technique has been used in numerous studies to test the thermal diffusivity of solid samples [14,22,23,24] and further progress has been made for data analysis, including finite difference and element schemes. Very limited research has been carried out, however, for thermal diffusivity measurements and analyses of granular materials such as powders used for additive manufacturing, or more generally, for heterogeneous materials.

Many engineering materials are heterogeneous at the macroscale and the concept of thermal diffusivity is then not as straightforward as for solid homogeneous samples. For example, when a heterogeneous material is made of two phases, solid particles and gas-filled space in between the particles, the thermal diffusivity values differ for both phases. An effective thermal diffusivity can be formally defined based on temperature differences at the bounding surfaces of the heterogeneous material. This effective or averaged thermal diffusivity depends not only on the volume fractions of the phases but on their arrangement, shape, or interface characteristics. At elevated temperatures the phases could react, adding to the difficulty in predicting the thermal diffusivity from imposed thermal conditions. If the concept of an effective thermal diffusivity is accepted for heterogeneous materials, the experimental approaches to measure thermal conductivity and diffusivity rely on raising the temperature in some sample regions of the sample and measuring the temperature changes with time elsewhere in the sample. Simple geometries help with experiments and data analyses. For example, using the transient hot wire approach, Wei et al. [1] examined the thermal conductivities of metal powders for powder bed additive manufacturing. In this approach, powder samples were placed in a copper holder and a platinum wire was immersed in it at the axial location. After applying heat, the resistance of the platinum wire was measured to determine the thermal conductivity of the powder sample. Andreotta et al. [2] experimentally determined the thermal conductivity of gas atomized IN718 powder particles using a TPS 2200 instrument (Transient Plane Source method). This method utilizes a spiral sensor which simultaneously heats up the sample and measures the time-dependent temperature changes. Zhang et al. [25] investigated the thermal conductivity of metal powder in LPBF AM for IN625 and Ti64 powders using a TA Instruments DLF 1200 laser flash instrument, and finite element (FE) heat transfer modeling. Ahsan et al. [26] determined the thermal diffusivity of IN718, Ti-6Al-4V and SS 304L metallic powders using the laser flash technique with a DXF 900 instrument. For their measurements, they used encapsulated powder samples in a holder with a lid. The laser flash system applied a laser pulse on the bottom of the sample and an infrared pyrometer measured the changes on the top of the sample surface. While LFD measurements of heterogeneous materials such as powders are not much more difficult than of solid samples, the analysis is oftentimes complicated because of foils that are added to keep the material in place in the sample holders. A common approach to measure liquid or powder samples with the LFD technique is to confine them in a holder with thin foils on top and bottom. These foils then become part of the measured system, which is effectively a three-layer system with the liquid or powder material as the middle layer. The problem of heat conduction of a three-layer system was examined analytically by Lee [27] and the analytical solution was used by Farooq et al. [28]. Ohta et al. [29] used Lee’s model to measure the thermal diffusivity of molten salt. Other solutions to the three-layer thermal conduction problem exist; Maeda et al. [30] used a logarithmic analysis proposed by James [31] to determine the thermal diffusivity of water and ethanol. A curve fitting method was performed in that study to estimate the thermal diffusivity value. All those studies on liquid thermal diffusivity using three-layered method showed comparison of measured data for reference samples with the available literature data. Different temperatures were used for the measurements.

The present study was conducted to investigate the thermal diffusivity of IN718 and Ti-6Al-4V powders using the LFD technique with a powder sample holder that effectively represents a three-layer system. Samples were measured over a temperature range and the experimental and analysis approaches were validated using reference samples with known thermal diffusivity values. The raw data were then analyzed with two model approaches: a Clark and Taylor analysis [17] that assumes a homogeneous sample and a three-layer model developed by Lee [27]. Previous reports on the powder bed thermal diffusivity of IN718 and TI-6Al-4V did not go into much detail about the raw data analysis and did not elaborate on the need to consider a multilayer analysis approach over assumptions of single-phase homogeneous samples. The current work demonstrates the inadequacy of treating raw data with a standard Clark and Taylor model while furthermore demonstrating the usefulness of multilayer model approaches to powder bed thermal diffusivity analysis.

## 2. Theory

### 2.1. Homogeneous Sample—The Parker Model

Parker et al. assumed a one-dimensional sample with length L, adiabatic boundary conditions, and an initial temperature distribution that is zero in the sample except for a thin surface layer, where a brief laser pulse is absorbed [13]. The relative temperature as a function of the Fourier number is given as:

(5) $$\frac{T}{T_{max}}=1+2\sum_{n=1}^{\infty }{\left(-1\right)}^{n}e^{-n^{2}\pi^{2}t^{*}}$$

where *t*^∗^ is the Fourier number:

(6) $$t^{*}=\alpha t/L^{2}\;$$

**Figure 1:**
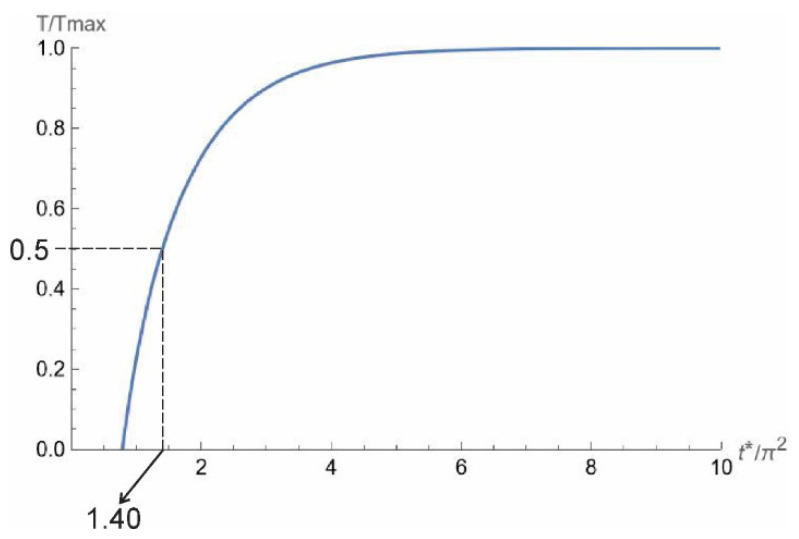
Normalized temperature vs. scaled time following Equation (1).

The relative temperature change with time is shown in Figure 1. Instead of time, the Fourier number times π^2^ is used for the x-axis. The relative temperature reaches half of the maximum value, i.e., 0.5, at π^2^*t*^∗^ = 1.40. In [13] a value is given of 1.38, but 1.40 is the value obtained from solving Equation (1). From the Fourier number, the thermal diffusivity can be determined from measured times at which the temperature signal reaches half of the maximum value, *t* = *t*_1/2_:

(7) $$\alpha =0.140\frac{L^{2}}{t_{1/2}}$$

### 2.2. Homogeneous Sample with Heat Loss Correction

Parker’s model assumed adiabatic boundary conditions. This assumption holds for near ambient temperature conditions that involve negligible radiative heat loss and for a quiescent atmosphere surrounding the samples that reduces the convective contribution to heat transfer. Adiabatic conditions furthermore require negligible contact between the sample and the sample holder to minimize heat conductive losses. With LFD equipment capable of measuring up to 2800 °C, the assumption of adiabatic boundary conditions must clearly be revised if high temperature measurements are performed. Cape and Lehman [19], Cowan [18], Watt [21], and Heckman [32] were among the first to include heat losses due to radiation or convection into their heat flow analyses. The Clark and Taylor analysis of radiative heat loss during LFD measurements is implemented in some of the commercial LFD equipment software and is used in this study [17]. The Clark and Taylor analysis falls within the group of analysis methods that use the ratio of times for the measurement signal to reach specific percentages of the signal maximum. used $\beta_{1}T_{max}$ and $\beta_{2}T_{max}$ as the levels reached by the thermogram in its rising part. The corresponding dimensionless times, Fourier numbers $t_{\beta_{1}}^{*}$ and $t_{\beta_{2}}^{*},$ depend on the heat losses. According to the definition of the Fourier number, the ratio $t_{\beta_{1}}^{*}/t_{\beta_{2}}^{*}$ is equal to the ratio of the corresponding times $t_{\beta_{1}}/t_{\beta_{2}}$. The thermal diffusivity is then obtained from the following equation:

(8) $$\alpha =t_{\gamma }^{*}\left(t_{\beta_{1}}/t_{\beta_{2}}\right)\frac{L^{2}}{t_{\gamma }}$$

where $t_{\gamma }^{*}$ is a function of $t_{\beta_{1}}/t_{\beta_{2}}$.

ASTM standard E1461-13 describes thermal diffusivity measurements with the laser flash method. The standard describes a correction method for thermal diffusivity based on the Clark and Taylor model [33]:

(9) $$\alpha_{c}=\alpha_{1/2}K_{R}/0.1388,$$

where $\alpha_{1/2}$ is the thermal diffusivity value calculated using the Parker formula. The correction factor *K_R_* is calculated using:

(10) $$K_{R}=-0.3461+0.3615\left(\frac{t_{0.75}}{t_{0.25}}\right)-0.0652{\left(\frac{t_{0.75}}{t_{0.25}}\right)}^{2}.$$

The ratios in Equation (5) are between the time for the measurement signal to reach 75% of its maximum and 25% of its maximum. The thermogram of the rear face is less affected by heat losses at short times after the heat pulse and at a smaller Biot number [29], [34]. Consequently, in the early time interval with the smaller Biot number, the thermogram is close to ideal curve for an adiabatic sample. In this case, Equation (9) with *Bi* = 0 can be applied at several partial times *t_β_*. In addition to radiative heat loss, external factors such as the stability of the temperature–response curve’s signal may also have an impact on the reported thermal diffusivity. Therefore, an appropriate approach for assessing thermal diffusion in solid materials at high temperatures must be developed that does not necessitate monitoring over a lengthy temperature–response time [34].

**Figure 2:**
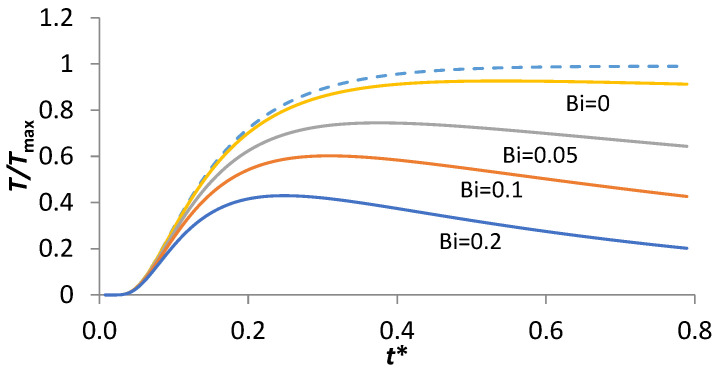
Normalized theoretical temperature–response curves with different Biot numbers (adapted from [34]).

The method of measuring thermal diffusivity from the rising part of the thermogram is associated with heat loss parameters or Biot numbers (Figure 2) [34]. The Biot number is defined as the ratio of thermal convection at surfaces of material to the thermal conduction of the material interior [35]. For significant radiative heat loss, thermal diffusivity can be expressed as [34]:

(11) $$\alpha =t_{\beta }^{*}\left(Bi\right)\frac{L^{2}}{t_{\beta }}$$

where $t_{\beta }$ is the time at which the thermogram reaches the level $\beta T_{max}$ in its rising part. The associated Fourier number $t_{\beta }^{*}$ depends on the heat losses (Biot number *Bi*). In the ideal case of an adiabatic sample (*Bi* = 0) and one dimensional heat flow, Equation (9) reduces to the original Parker equation with $t_{1/2}^{*}$ = 0.1388.

### 2.3. Heterogeneous Samples-Three-Layered Analysis

**Figure 3:**
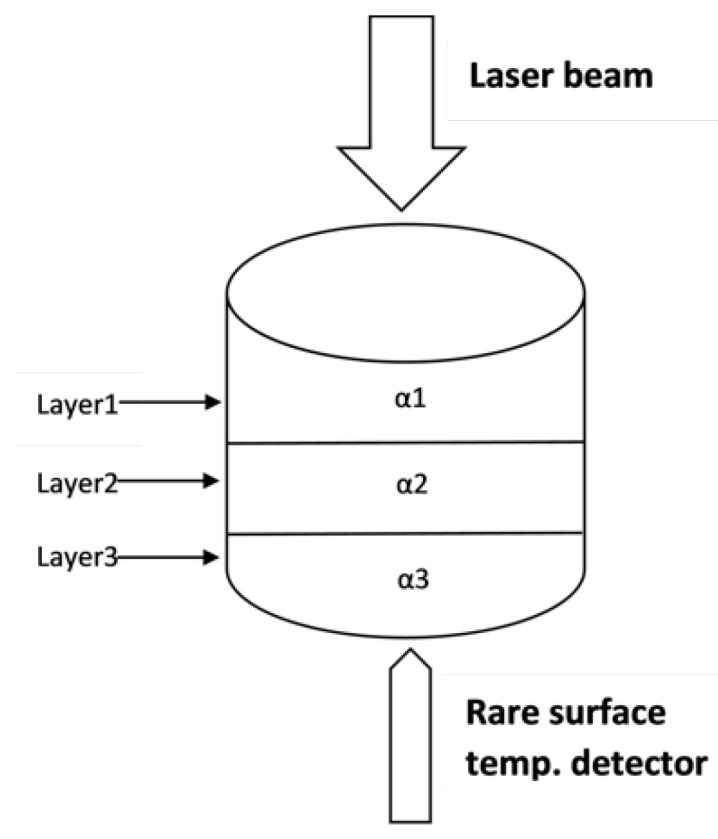
Three-layered sample.

The early models for determining thermal diffusivity from LFD measurements were developed for homogeneous samples. A theoretical model for measuring the thermal diffusivity of layered and dispersed sample was developed by Lee [27]. The principle of the three-layered sample is illustrated in Figure 3.

Lee’s model is based on the assumptions that each layer is homogeneous, heat flow is one dimensional, no heat loss on the sample surface and no interfacial thermal contact resistance. The simplified equation of temperature rise on the rear face of the three-layered sample after the pulse is [27]:

(12) $$T\left(L_{1}+L_{2}+L_{3},t\right)=\;T_{max}\left[1+2\overset{\infty }{\underset{n=1}{\sum }}\frac{\left(\omega_{1}\chi_{1}+\omega_{2}\chi_{2}+\omega_{3}\chi_{3}\right)exp{\left[-{\left(i^{2}\pi^{2}\right)}^{2}t_{i}^{*}\right]}^{}}{\omega_{1}\chi_{1}\cos\left(\gamma_{n}\omega_{1}\right)+\omega_{2}\chi_{2}\cos\left(\gamma_{n}\omega_{2}\right)+\omega_{3}\chi_{3}\cos\left(\gamma_{n}\omega_{3}\right)+\omega_{4}\chi_{4}\cos\left(\gamma_{n}\omega_{4}\right)}\right]$$

where

$$\chi_{1}=H_{1/3}\eta_{3/1}+H_{1/2}\eta_{2/1}+H_{2/3}\eta_{3/2}+1,$$

$$\chi_{2}=H_{1/3}\eta_{3/1}-H_{1/2}\eta_{2/1}+H_{2/3}\eta_{3/2}-1,$$

$$\chi_{3}=H_{1/3}\eta_{3/1}-H_{1/2}\eta_{2/1}-H_{2/3}\eta_{3/2}+1,$$

$$\chi_{4}=H_{1/3}\eta_{3/1}+H_{1/2}\eta_{2/1}-H_{2/3}\eta_{3/2}-1,$$

$$\omega_{1}=\eta_{1/3}+\eta_{2/3}+1,$$

(13) $$\omega_{2}=\eta_{1/3}+\eta_{2/3}-1,$$

$$\omega_{3}=\eta_{1/3}-\eta_{2/3}+1,$$

$$\omega_{4}=\eta_{1/3}-\eta_{2/3}-1,$$

and γ is positive roots of the following characteristic equation:

(14) $$\chi_{1}\cos\left(\gamma \omega_{1}\right)+\chi_{2}\cos\left(\gamma \omega_{2}\right)+\chi_{3}\cos\left(\gamma \omega_{3}\right)+\chi_{4}\cos\left(\gamma \omega_{4}\right)=0.$$

H*_i_* = volumetric heat capacity, $\eta_{i}$ = square root heat diffusion time of the *i*-th layer, and i=1,2,3.

Then, the Fourier number of each layer is

(15) $$t_{i}^{*}=\frac{\alpha_{i}t_{1/2}}{L_{i}^{2}}=\frac{t_{1/2}}{\eta_{i}^{2}}$$

This study used the three-layered model for analyzing the thermal diffusivity of the IN718 and Ti-6Al-4V powders.

## 3. Experimental Apparatus and Specimen

**Figure 4:**
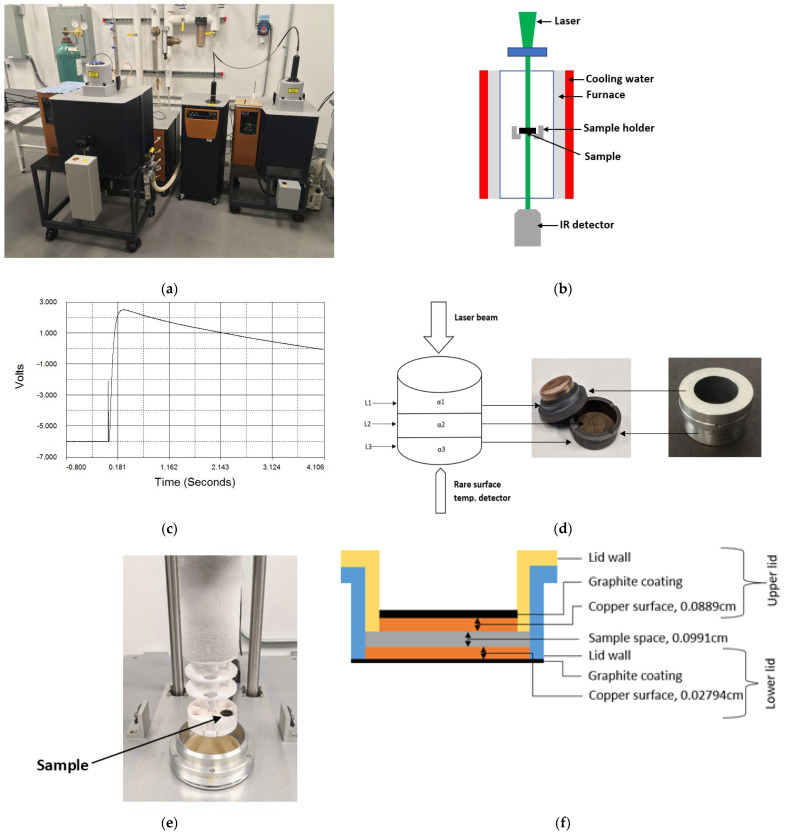

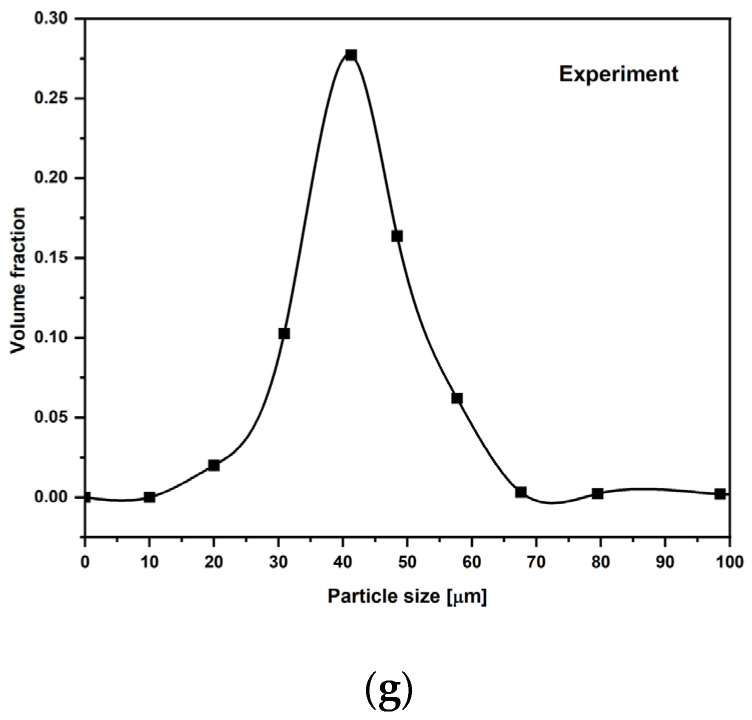
(**a**) DLF 1600 Laser Flash apparatus; (**b**) schematic of laser flash method; (**c**) typical thermogram generated by the instrument software; (**d**) powder sample holder and tri-layer configuration; (**e**) powder cell loading in alumina holder; (**f**) cross-sectional view of sample holder; (**g**) size distribution curve of IN718 powder.

A TA Instruments laser flash diffusivity instrument (TA instruments, New Castle, DE 19720, USA) was used for this study (Figure 4a). The instrument consists of the DLF 1600 laser source and the EM 1600 furnace, which contains an alumina sample holder carousel. This instrument uses a top-hat shaped laser pulse to heat the top surface of samples within the furnace. The rise in temperature at the bottom of the sample is recorded using an infrared camera. The schematic of the laser flash method is shown in Figure 4b. The equipment software determines the thermal diffusivity from the measured thickness of the samples and the voltage signal of the infrared camera recorded as a function of time, which is considered to be proportional to the temperature rise in the sample. A special sample holder shown in Figure 4d, was used to perform the measurements on powder samples in this study. This powder cell has an upper and a lower copper foil. The inner diameter of the cell is 1.5875 cm and inner gap height 0.0991 cm. A thin layer of the test specimen is contained between the copper foils. The specimens must touch the copper foils to establish good thermal contact between sample and copper foils. The external top and bottom copper foil surfaces are coated with graphite to improve the absorption of the laser. The sample holder is then inserted into an alumina holder which is built into the system, as shown in Figure 4e.

The cross-sectional view of the powder holder is shown in Figure 4f. Solid samples were also measured with the standard solid sample holder, which requires samples between 12.4 and 12.7 mm in diameter and with typical thicknesses of 1.2–2.0 mm. The solid metal samples were slightly ground with 600 grit SiC paper in order to improve the laser absorption. Additionally, a graphite coating was sprayed onto both sample surfaces to improve the laser energy absorption into the metal surfaces. Measurements were made in a slow flowing nitrogen gas atmosphere inside the furnace at a very slight overpressure. The purity of nitrogen gas was 99.998%. After loading the sample, the furnace was pumped down to below 0.05 torr and backfilled with nitrogen gas. A representative voltage signal as a function of time is shown in Figure 4c. Following the brief laser pulse that slightly raises the temperature on the top of the sample holder, the temperature rises at the bottom and then falls off again. Measurements were performed with IN718 powder.

The IN718 powder used for this study had a powder particle size distribution between 20 µm and 70 µm; the Ti-6Al-4V powder was LPBF powder with the same nominal size distribution. Both powders were gas-atomized with spherical particle shapes. The powder was poured into the powder holder. Attempts to manually compact the powder in the powder holder did not reveal any effect on the measured thermal diffusivity values. Powder particle size distribution curve of IN718 powder is shown in Figure 4g.

## 4. Results and Discussion

The measurements were performed in two steps. In the first step, solid reference samples were measured with the standard solid sample holder. The standard solid sample holder is used for solid samples only. This first step was taken to ensure that the overall approach yielded acceptable results for known samples and for the standard sample holder. The overall approach includes the raw data analysis using the Clark and Taylor model and the IR detector that generates the raw data, i.e., the IR voltage signal at the bottom of the sample as a function of time. The special sample holder is shown in Figure 4d,f and is specifically designed for use with powder or liquid samples. In the second step the special sample holder was used with IN718 and Ti-6Al-4V powder samples to obtain thermal diffusivity data.

### 4.1. Solid Reference Samples, Standard Solid Sample Holder

The thermal diffusivity values were measured of solid IN718, Ti-6Al-4V, and 304 stainless steel.

The mean experimental thermal diffusivity of the reference samples at various temperatures is presented in Table 1 along with literature data for the same alloys. The data shown for the experiments in Table 1 were obtained applying the Clark and Taylor model to the measured data. Agazhanov [36] used the laser flash method with a cylindrical shaped IN718 solid sample. Milosevic [37] used a thin disk of solid Ti64 samples in their laser flash measurement of the thermal diffusivity. Sweet [24] used a disk-shaped sample with 1.27 cm diameter and the laser flash method for their measurement. The three publications do not indicate how the thermal diffusivity values were averaged over the temperature intervals and what model was used to determine the thermal diffusivity values. In all three studies measurements were performed in an argon furnace environment. Although nitrogen gas was used in the current study, the results are comparable with the literature cited above because argon and nitrogen gas have similar thermal diffusivity [38]. The deviation of experimental data in this study from the literature is between 4.0% and 6.5%.

### 4.2. Powder Sample, Special Sample Holder

**Figure 5:**
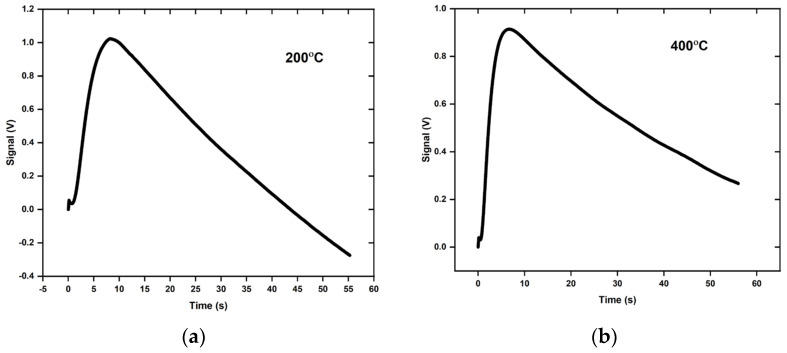

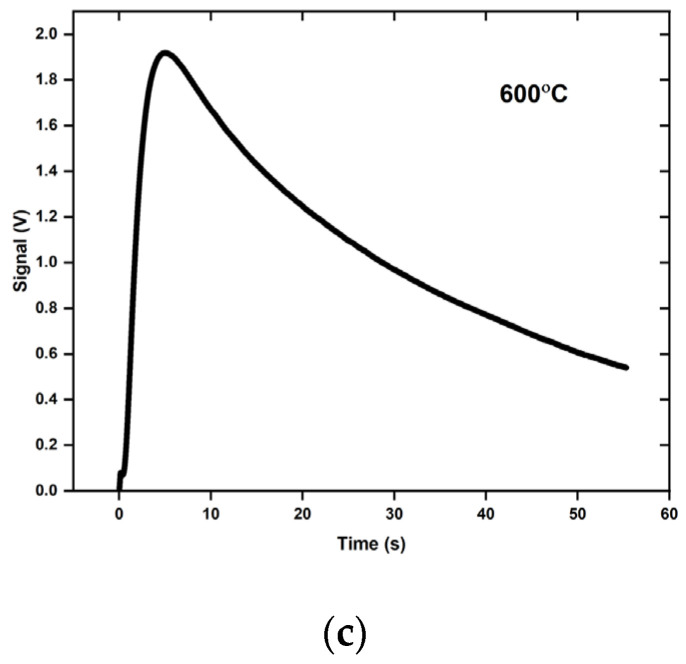
Thermogram from a DLF1600 instrument at 200 °C (**a**), 400 °C (**b**), and 600 °C (**c**) for IN718 powder sample.

The thermal diffusivity of IN718 and Ti-6Al-4V powder was measured with the special sample holder at different temperatures. Figure 5 shows the direct measurement results at temperatures of 200 °C, 400 °C, and 600 °C. The relatively steep drop in the IR detector voltage signal after the peak indicates heat loss of the sample. The Clark and Taylor model was first used to analyze the measured data as if the tri-layer system was effectively one homogeneous sample. The assumption of a homogeneous sample and the Clark and Taylor model are significantly easier to apply than models based on a multilayer arrangement. The results are summarized in Table 2.

The thermal diffusivity values of the IN718 powder are on the order of 4 × 10^−4^ cm^2^/s when the Clark and Taylor model is applied. According to references [26,39], the average thermal diffusivity of IN718 powder over temperature ranges from 26.85 °C to 526.85 °C and from 100.4 °C to 550.2 °C is 1.5 × 10^−3^ cm^2^/s and 1.6 × 10^−3^ cm^2^/s, respectively. The strong deviation between the literature results and the Clark and Taylor analysis of an assumed pseudo homogeneous tri-layer system shows that a more refined approach is necessary to analyze the raw data, i.e., the temperature or voltage vs. time curves than the Clark and Taylor model. This refined approach should take the tri-layer arrangement explicitly into account. The experimental data and the deviation from reported values clearly reveal the inadequacy of a homogeneous sample assumption. The same conclusion can be reached with a theoretical assessment developed by Kerrisk [40].

**Figure 6:**
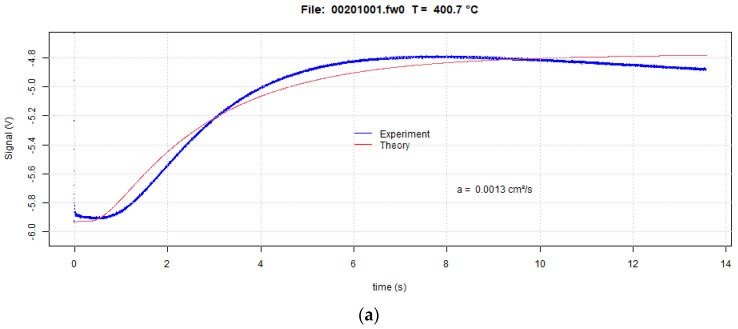

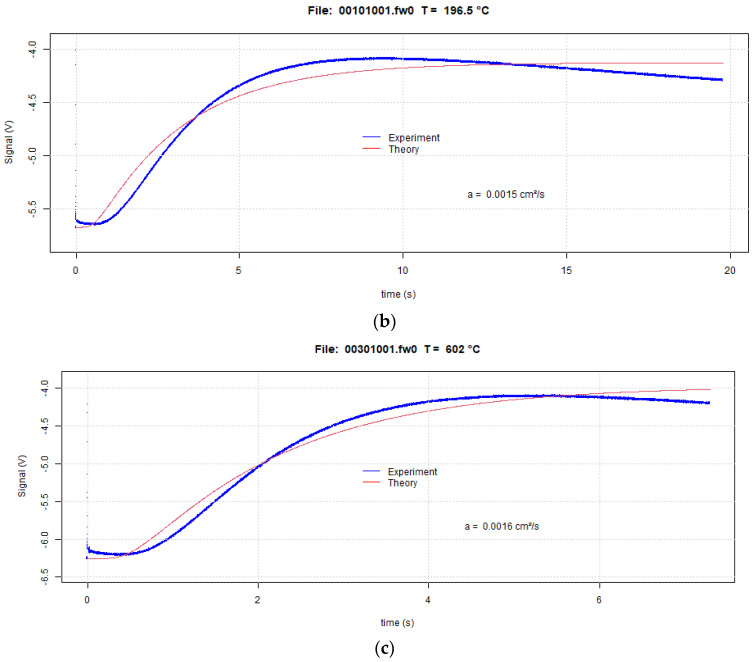
Thermograms for IN718 powder at 200 °C (**a**), 400 °C (**b**) and 600 °C (**c**) temperatures with reduced temperature–response time.

The analytical model presented by Lee [27] for heat flow in a tri-layer system requires the specific heat and density of all three layers to be known over the temperature range of interest. In addition, the thermal diffusivity values must be known for two of the three layers with the thermal diffusivity of the third layer as the only unknown. In the current work, the top and bottom layers were made of thin Cu foil while the powder bed made up the center layer. Figure 6 shows raw data along with predicted curves following Lee’s analysis for IN718 powder at 200 °C, 400 °C, and 600 °C temperatures for the heating portions of the heat pulse. The heating portion is used because Lee’s analysis uses the heating and not the cooling portion. The blue curves depict the experimental curve while the red curves represent the predicted signal based on Lee’s model. The experimental voltage values decrease after the peak maximum while the theoretical curve remains constant. Lee’s tri-layer model assumes adiabatic conditions, which explains the constant voltage and hence temperature after the signal reaches the maximum. The experimental sample transfers energy into the surrounding, which contributes to the signal decrease and hence cooling after the maximum. At the beginning of the curve, the voltage remains constant after the laser pulse that is at zero time for a brief amount of time. During that brief time, the energy imparted onto the sample surface by the laser flash diffuses through the sample to the bottom of the sample that the IR camera probes and after a transient stage then raises the temperature of the sample bottom. Table 3 shows the thermal diffusivity values (α) of IN718 powder using Lee’s three-layered analysis at different temperatures. The results from the three-layer analysis in this work deviate from the literature values by between 2.4% and 7.1%, which indicates good agreement. The raw data voltage curves coincide for the three shots that the laser flash instrument took at each temperature. Errors in the thermal diffusivity results enter in addition to the negligible error in the raw data variation for each temperature mainly in the uncertainty of the material property data that is needed to complete the three-layer analysis. Since the density, specific heat, and thermal diffusivity values are required for the copper top and bottom foils and the density and specific heat of the powder layer, the powder layer thermal diffusivity accuracy depends on the accuracy of those input parameters. The current measurements, very similar to the literature values, reflect a decrease in the thermal diffusivity values at 400 °C compared to the values at 200 °C and 600 °C. This unusual behavior resembles the change in thermal conductivity reported for pure nickel and dilute nickel solid solutions [41] where the thermal conductivity decreases up to the Curie temperature and then increases again. However, the same behavior was not observed for nickel-based superalloys [41]. Specific heat measurements of IN718 also do not show an extremum during heating, which could explain an extremum in the thermal diffusivity if the thermal conductivity is monotonous with temperature increase [36]. Further research is necessary to identify the thermal diffusivity dip during the heating of the IN718 powders. It is conceivable that the gas atomization of the powders and the concomitant fast cooling induces nonequilibrium effects such as extended solubilities, formation, or suppression of phases that affect the thermal response of the powders.

**Figure 7:**
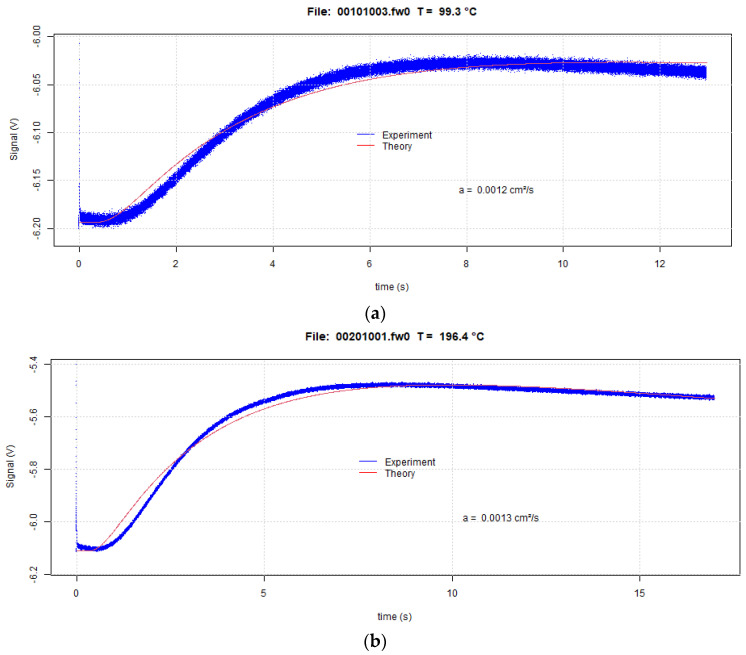
Thermograms for Ti-6Al-4V powder sample at 100 °C (**a**) and 200 °C (**b**) with reduced temperature–response time.

Figure 7 shows thermograms for Ti-6Al-4V powder at 100 °C and 200 °C. Table 4 shows the corresponding thermal diffusivity values (α) after applying Lee’s three-layer model. The voltage signals and the calculated signals based on Lee’s model show reasonable agreement during the heating portion of the signal (increasing voltage values). A very good match should not be expected because Lee’s three-layer model assumes adiabatic sample conditions, another important assumption concerns the thermal contact resistance between the copper foils and the central powder layer that is assumed to be zero. Despite these assumptions, the thermal diffusivity values from this work are in good agreement with the literature values.

The powder bed thermal diffusivity values for IN718 and Ti-6AL-4V are more than one order of magnitude smaller than those of the solid samples. For solid IN718, Agazhanov reported values between 0.03 and 0.04 cm^2^/s [36], for Ti-6Al-4V, the values are similar, approximately between 0.026 and 0.032 cm^2^/s [42]. Attempts to manually compact the powder in the special sample holder did not change the measured thermal diffusivity values. Another important aspect of thermophysical property measurements with powder beds is their behavior with increasing temperatures. At elevated temperatures, powder sintering can complicate the interpretation of measured data. Decreasing gas pore volumes and increasing solid fractions with sintering increase the conduction component of thermal conduction over convection or radiation contributions. In light of the widespread use of powder beds in additive manufacturing, the results obtained in this work highlight the more than order of magnitude decrease in the thermal diffusivity of powder beds over solid samples. The solidification of melt-pools during additive manufacturing depends on the rate at which energy liberated at the liquid–solid interface is transported away from the interface. Reduced thermal diffusivities in powder beds therefore affect the energy transport rate away from the interface into the solid and indirectly therefore affect the solidification behavior. The current work furthermore shows a need for multilayer models of heat transfer. Radiation and convection boundary conditions should in the future be incorporated into these models, but the threshold in analytical complexity might be reached where numerical models and finite difference methods prove more tractable.

## 5. Conclusions

The thermal diffusivity was determined of IN718 and Ti-6Al-4V powder beds as a function of temperature and using the laser flash method. The raw data consist of a voltage signal as a function of time that corresponds to the temperature increase at the bottom of samples after the sample top is exposed to a laser flash. The comparison between data analysis assuming a homogeneous sample and the Clark and Taylor model and a more complex tri-layer model shows the need for the tri-layer analysis. Powder bed thermal diffusivity values are over an order of magnitude smaller than the values of solid samples of the same alloy. The thermal diffusivity behavior of IN718 powder beds resembles the behavior of pure Ni or dilute alloys of Ni with a thermal diffusivity dip at approximately 400 °C. This dip is not observed in solid IN718 and points toward possible implications of the gas atomization process on powder characteristics. These findings suggest promising opportunities for further research, including exploring the underlying mechanisms of the observed thermal diffusivity dip, potential optimizations in additive manufacturing processes for IN718 and similar materials, and correlation analysis between thermal diffusion performance and the microstructure of the LPBF-printed components.

## Figures and Tables

**Table 1:** Thermal diffusivity of solid reference samples. The deviation is the difference between the measured and the literature values divided by the literature value.

**Samples**		**Mean Thermal Diffusivity (cm^2^/s)**	**Deviation**
In718 solid	Experiment (Temp. range: 100 °C–600 °C)	0.0434	4.8%
	Agazhanov [36] (Temp. range: 126.85 °C–626.85 °C)	0.0414	
Ti64 solid	Experiment (Temp. range: 100 °C–600 °C)	0.0362	4.0%
	Milosevic [37] (Temp. range: 48.85 °C–584.85 °C)	0.0377	
Stainless steel solid	Experiment (Temp. range: 50 °C–400 °C)	0.0461	6.5%
	Sweet [24] (Temp. range: 50 °C–400 °C)	0.0433	

**Table 2:** Calculation of thermal diffusivity by the Clark and Taylor formula from the thermograms in Figure 5.

Temperature (°C)	Max. Temp. Signal (V)	t_0.75_ (s)	t_0.25_ (s)	t_0.5_ (s)	α (cm^2^/s)
200	1.02	2.36	1.1	3.2	0.00040
400	0.91	2.97	1.4	3.09	0.00041
600	1.92	2.4	1.1	2.1	0.00062

**Table 3:** Thermal diffusivity of IN718 powder and literature values.

Temperatures (°C)	α Measured (cm^2^/s)	**α from** Literature (cm^2^/s) [26,39]	Deviation
200	0.0015	0.00159	5.7%
400	0.0013	0.0014	7.1%
600	0.0016	0.00164	2.4%

**Table 4:** Thermal diffusivity of Ti-6Al-4V powder sample from current work and from the literature [25].

Temperatures (°C)	**α (cm^2^/s)**	α from Literature (cm^2^/s) [25]
100	0.0012	0.00143
200	0.0013	0.00168

## Data Availability

Data is unavailable due to privacy or ethical restrictions.
